# Exploring Myocardial Ischemia-Reperfusion Injury Mechanism of Cinnamon by Network Pharmacology, Molecular Docking, and Experiment Validation

**DOI:** 10.1155/2023/1066057

**Published:** 2023-02-23

**Authors:** Tao Xue, Yan Xue, Yangyue Fang, Chuanghong Lu, Yu Fu, Zefeng Lai, Xiaojun Qin, Feng Huang, Zhiyu Zeng, Jianping Huang

**Affiliations:** ^1^Alibaba Business School, Hangzhou Normal University, Hangzhou 310000, China; ^2^Department of Cardiology, The First Affiliated Hospital of Guangxi Medical University, Nanning 53000, China; ^3^Guangxi Key Laboratory of Precision Medicine in Cardio-Cerebrovascular Diseases Control and Prevention, Nanning 53000, China; ^4^Guangxi Clinical Research Center for Cardio-Cerebrovascular Diseases, Nanning 53000, China; ^5^Pharmaceutical College, Guangxi Medical University, Nanning 53000, China; ^6^Department of Geriatric Cardiology, The First Affiliated Hospital of Guangxi Medical University, Nanning 53000, China

## Abstract

Myocardial ischemia-reperfusion injury (MIRI) is a common complication of acute myocardial infarction that seriously endangers human health. Cinnamon, a traditional Chinese medicine, has been used to counteract MIRI as it has been shown to possess anti-inflammatory and antioxidant properties. To investigate the mechanisms of action of cinnamon in the treatment of MIRI, a deep learning-based network pharmacology method was established to predict potential active compounds and targets. The results of the network pharmacology showed that oleic acid, palmitic acid, beta-sitosterol, eugenol, taxifolin, and cinnamaldehyde were the main active compounds, and phosphatidylinositol-3 kinase (PI3K)/protein kinase B (Akt), mitogen-activated protein kinase (MAPK), interleukin (IL)-7, and hypoxia-inducible factor 1 (HIF-1) are promising signaling pathways. Further molecular docking tests revealed that these active compounds and targets exhibited good binding abilities. Finally, experimental validation using a zebrafish model demonstrated that taxifolin, the active compound of cinnamon, has a potential protective effect against MIRI.

## 1. Introduction

Acute myocardial infarction (AMI) is a life-threatening cardiovascular condition that has significant global health and economic effects [1]. For example, in China, the mortality rate of AMI has been steadily rising since 2002, reaching 62.33 per 100,000 in urban regions and 78.47 per 100,000 in rural areas in 2018 [2]. In the United States, more than 11 million people were hospitalized with AMI in 2010, which resulted in a direct economic cost of more than $450 billion [3].

AMI is frequently accompanied by myocardial ischemia-reperfusion injury (MIRI), which can exacerbate cardiac dysfunction and increase the likelihood of a poor prognosis for AMI [4]. It is known that various pathophysiological factors are involved in MIRI [5], such as oxidative stress, apoptosis of cardiomyocytes, calcium overload, endothelial dysfunction, mitochondrial dysfunction, and intramyocardial inflammation [6]. Although the understanding of the mechanism of MIRI has made great progress over the past decade, the clinical therapeutic effect appears to be limited. Therefore, there is an urgent need to identify new targets and transformative therapeutic measures. In recent years, traditional Chinese medicine (TCM) has been used to counteract MIRI [7–9].

Cinnamon, a commonly used spice worldwide, is considered to have important pharmacological value and was included in the Chinese Pharmacopoeia in 2020. Cinnamon has antioxidant, anti-inflammatory, endothelium protecting, and immune response regulating properties and has been used in preclinical and clinical research to prevent and cure cardiovascular disorders [10]. Moreover, cinnamon and its extracts have been shown to have the potential to aid in the treatment of MIRI by reducing the area of myocardial infarction via various mechanisms [11, 12]. These studies highlight the possible relevance of cinnamon in MIRI therapy, but its mechanism of action remains unexplained.

Network pharmacology is a new technology that integrates chemistry, pharmacology, and bioinformatics to provide new insights into the complex mechanisms of action of herbal medicines [13]. However, some challenges with the network pharmacology approach remain, such as the lack of comprehensive data on various drugs, genes, and proteins [14]. Nevertheless, drug-target interaction prediction (DTI), as one of the most direct and successful methods for discovering new drugs and targets, can help to some extent solve the problems of network pharmacology. And several new DTI methods have been developed in recent years, all of which have shown promising results [15, 16]. In addition, molecular docking, as a tool for predicting binding patterns between small molecules and target proteins, is an important bridge between structural chemistry and the life sciences.

**Figure 1:**
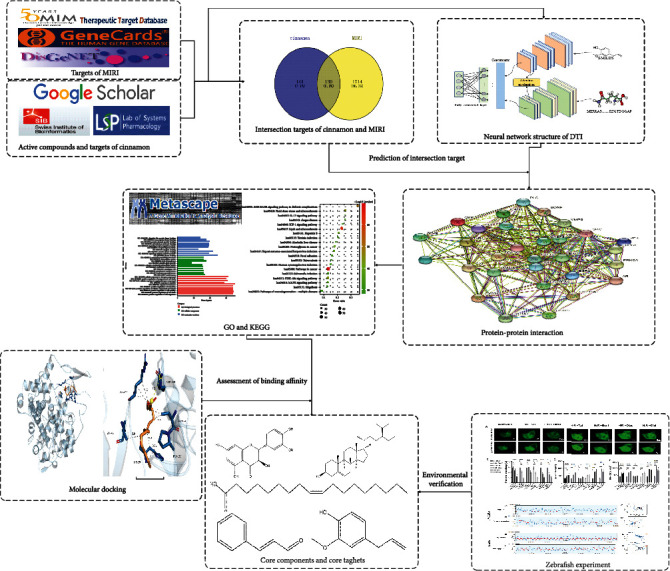
The main workflow. It deciphers the exploration process of cinnamon on MIRI using the network pharmacology approach, which involves deep learning, molecular docking, and experimental validation.

In this study, to better examine the primary components and probable mechanisms of cinnamon in the treatment of MIRI, a deep learning-based network pharmacology technique, molecular docking, and experiment were provided (Figure 1).

## 2. Materials and Methods

### 2.1. The Collection and Screening of the Active Compounds of Cinnamon

The chemical compounds of cinnamon were taken from the TCM System Pharmacology (TCMSP) Database (http://tcmspw.com/tcmsp.php) [17], following a screening process based on an oral bioavailability (OB) of ≥30% and drug-likeness (DL) of ≥0.18. As a supplement, additional active compounds were added by reviewing the literature.

### 2.2. Collection of Targets of Cinnamon for the Treatment of MIRI

The targets of cinnamon were obtained from the TCMSP and SwissTargetPrediction [18], and the targets for MIRI were found by scanning the Online Mendelian Inheritance in Man (OMIM) [19], the Therapeutic Target Database (TTD) (http://db.idrblab.net/ttd/) [20], the GeneCards database (http://www.genecards.org) [21], and the DisGeNET database (http://www.disgenet.org/web/DisGeNET/) [22] using the phrase “ischemia-reperfusion injury.” The active compounds of cinnamon and the targets of MIRI were then imported into the online tool Venny (http://bioinfogp.cnb.csic.es/tools/venny/), from which the intersection targets could be outputted.

### 2.3. Predictions of Targets Based on DTI

DTI has recently been shown to be effective in identifying potential targets. In 2022, Zhao et al. [23] proposed HyperAttentionDTI, which is a DTI-based model that integrates sequence-based deep learning and attention mechanisms. In order to display the interactions between amino acids and atoms and to manage how features are represented on the channel, HyperAttentionDTI calculates an attention vector for each amino acid and atom pair. In this study, HyperAttentionDTI was used for prediction due to its greater performance when compared to other models.

Based on the active compounds and targets collected, compounds were transformed into the simplified molecular input line entry system (SMILES) strings, the targets were converted into protein amino acid sequences, and each SMILES string was matched to each amino acid sequence one by one. We collected 29063 items of data as a test set. And the Davis dataset is a drug-target interaction dataset composed primarily of the SMILES structures of compounds, amino acid sequences of proteins, and interaction labels (0 or 1). In this study, the model was trained using five-fold cross-validation on the Davis dataset. Finally, based on the results of the HyperAttentionDTI predictions, the number of intersection targets was increased, providing a rich database for the subsequent analysis step of the network pharmacology method.

### 2.4. Construction of the Compound-Disease-Target Network

The active compounds and potential therapeutic targets were imported into Cytoscape (version 3.9.1) [24] to create a compound-disease-target network. Then, Network Analyzer was used to identify the potential main compounds of cinnamon for the treatment of MIRI during network visualization.

### 2.5. Construction of the Protein-Protein Interaction (PPI) Network

The intersection targets were loaded into the Search Tool for the Retrieval of Interacting Genes/Proteins (STRING) database (https://string-db.org) [25] to obtain the PPI network, with the species set to “*Homo sapiens*” and the confidence score set to ≥0.7. The network was imported into Cytoscape for visualization and key target screening. Degree unDir, betweenness unDir, and closeness unDir were three crucial metrics determined by Centiscape (version 2.2) that were chosen as screening criteria based on the PPI network.

### 2.6. Enrichment Analysis

Gene Ontology (GO) functional enrichment analysis and Kyoto Encyclopedia of Genes and Genomes (KEGG) metabolic pathway enrichment analysis (*P* < 0.01) were performed on core targets using Metascape (https://metascape.org/) [26]. The species was set as *H. sapiens*, and the Bioinformatics website (http://www.bioinformatics.com.cn/) and the R toolkit (version 4.1.2) were used to draw the graph. Then, a cinnamon-active compound-target-pathway network was built, which was required for further therapeutic target screening.

### 2.7. Molecular Docking Verification

To further verify the main compounds and targets outlined above, molecular docking was used to calculate their binding affinities. First, the crystal structures of the protein targets and related information were obtained from the UniProt [27] (https://www.uniprot.org/) and RCSB Protein Data Bank (PDB) [28] (https://www.rcsb.org/) databases. For proteins that could not be queried, a PDB format file was created using a homology modeling method, and a three-dimensional (3D) structure was predicted using Aphafold2 [29]. Then, using AutoDockTools (version 1.5.7), operations, such as hydrogenation, charge addition, removal of water molecules, and removal of metal ions, were carried out, and the files were ultimately converted into pdbqt format. Second, the 3D structures of the active compounds were received from the PubChem [30] database (https://pubchem.ncbi.nlm.nih.gov/). Compounds in which the 3D structure could not be found were transformed into a 3D structure according to their two-dimensional structure. Subsequently, qvina-w was used to perform blind docking while AutoDockTools was used to create the global docking box [31]. The binding score was used to evaluate the ability of a natural compound to bind to the target. Finally, Python (version 3.9.1) and Pymol (version 2.4) were used to create heat maps and 3D docking maps of the docking results.

### 2.8. Animal Experiment

We designed a zebrafish model to confirm the protective effect of the obtained composition on MIRI. Due to equipment and financial constraints, only oleic acid, eugenol, taxifolin, and cinnamaldehyde were selected for the experiment. We randomly divided 84 zebrafish larvae into 7 groups and pretreated them with different microinjections 12 hours before hypoxia and reoxygenation (H/R). We then assessed their cardiac function and arrhythmia.

#### 2.8.1. Chemicals

We obtained 3,3′-dimethoxybenzidine from Aladdin (Shanghai, China), and 1-phenyl 2-thiourea (PTU) was purchased from Sigma-Aldrich (St. Louis, MO).

#### 2.8.2. Animal Care and Use

We used the AB wild-type strain and the transgenic line, Tg (cmlc:EGFP), of zebrafish (purchased from Guangxi Yisheng Biotechnology Co., Ltd. Nanning, GX, China). Zebrafish were housed in the zebrafish husbandry center of Guangxi Medical University, where they were fed with live brine shrimp twice daily and maintained at 28.5°C on a 14-hour light/10-hour dark cycle. As described in a previous study [32], 0.3% PTU was added to the egg water of the Tg (cmlc:EGFP) fish 24 hours postfertilization (hpf), and fluorescent microscopy was used to select healthy embryonic zebrafish with fluorescent hearts at 48 hpf. All procedures were approved by the Guangxi Medical University Animal Care and Use Committee.

We randomly assigned 84 larvae to 7 groups. They were pretreated with Phosphate Buffer Solution (PBS, purchased from Sigma-Aldrich, St. Louis, MO), 1% DMSO (purchased from Sigma-Aldrich, St. Louis, MO), eugenol (0.1 *μ*g/g body weight, purchased from Sigma-Aldrich, St. Louis, MO), cinnamaldehyde (0.5 *μ*g/g body weight, purchased from Sigma-Aldrich, St. Louis, MO), oleic acid (1.5 *μ*g/g body weight, purchased from Sigma-Aldrich, St. Louis, MO), and taxifolin (0.5 *μ*g/g body weight, purchased from Sigma-Aldrich, St. Louis, MO) by microinjection 12 hours before H/R or normoxic water starting at 2 days post-fertilization (dpf), respectively.

#### 2.8.3. Optical Imaging and Heart Function Analysis

The environmental temperature was maintained at 23°C ± 2°C. Hearts were examined between 2 and 6 dpf, when a distinct ventricle and atrium were present. To evaluate cardiac function, the Tg (cmlc:EGFP) zebrafish embryos were embedded in 4% methylcellulose (warmed to room temperature) and subjected to video capturing, maintaining the anterior orientation to the left and the dorsal orientation to the top of the field. Direct immersion optics were used in conjunction with a digital high-speed camera (100 frames/second; C13440; Hamamatsu Digital Camera) mounted on a Leica microscope (DMi 8; McBain Instruments) to record 15-second movies of beating hearts. Images were captured using the HC Image software (Hamamatsu). Cardiac function was analyzed from the high-speed movies using a semiautomatic optical heartbeat analysis software (freely available for research purposes at http://www.sohasoftware.com), which quantifies heart rate (HR), diastolic area (DA)/systolic area (SA), and fractional area change (FAC). HR variability (HRV) was analyzed from the recorded videos. Image analysis software Ethovision XT (Noldus Information Technology, Inc., Leesburg, VA) was used to automate the procedure for counting the HR. Data from each record were exported and archived in text format for further HRV analysis. Nonlinear analysis provided two consecutive RR intervals (RRn and RRn +1), which were projected on the Poincaré graph and used to adjust to an elliptical function.

#### 2.8.4. Statistical Analysis

All results are shown as means ± standard deviations. Comparisons between groups were evaluated using one-way analyses of variance and Student's *t*-tests. Statistical analyses were performed using Prism version 6.01 (GraphPad Software, San Diego, California, CA). Data were considered statistically significant at *P* < 0.05.

## 3. Results

### 3.1. Active Compounds and Potential Targets of Cinnamon

To investigate the therapeutic mechanism, we first collected the active ingredients of cinnamon. We retrieved 220 cinnamon compounds from the TCMSP database. After screening with an OB of 30% and DL of 0.18, 7 compounds remained. As a supplement, 12 additional active compounds were incorporated by analyzing the literature [33, 34] (Table 1).

The corresponding targets of the active compounds of cinnamon were obtained from the TCMSP and SwissTargetPrediction databases, and 271 targets were identified after merging the UniProt database entries and deleting duplicate values. In addition, 1844 targets of MIRI were obtained by screening and de-duplicating the GeneCards database, the TTD database, the OMIM database, and the DisGeNET database. After screening 271 active compound targets and 1844 MIRI targets using the Venn diagram, 130 intersection targets remained.

### 3.2. Predictions of the DTI Model

Since DTI is a binary classification task, the accuracy, precision, recall, and area under the curve (AUC) metrics were used to assess the performance of the model. The results are shown in Table 2. HyperAttentionDTI revealed 4214 active compound-target pairs with interaction relationships. The number of targets that the model predicted for each compound is displayed in Table 3. These targets were then combined with the intersecting targets obtained in Section 3.1 and deweighted to produce 1144 targets (Supplementary Table S1).

### 3.3. Construction of the Compound-Disease-Target Network

**Figure 2:**
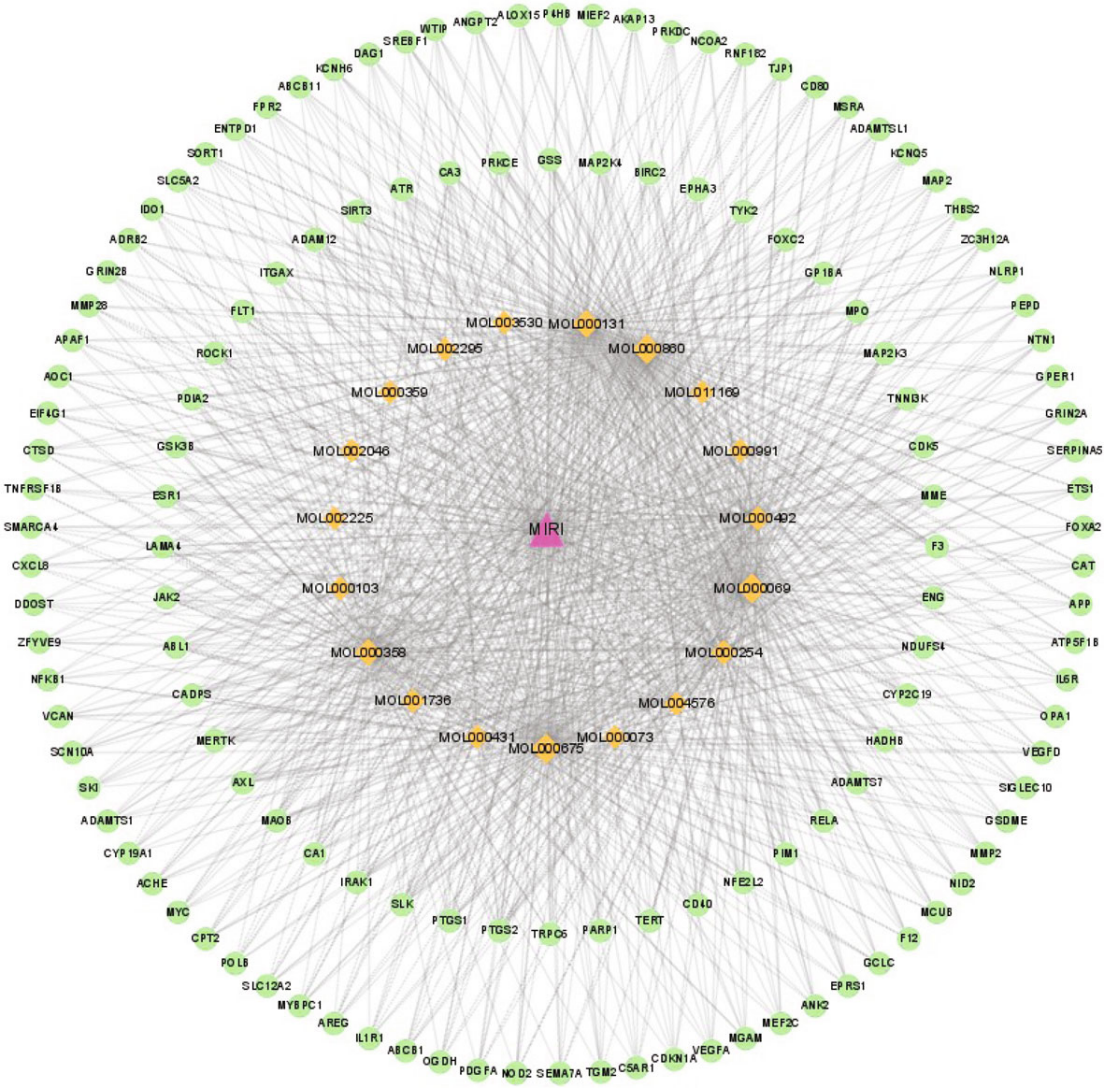
The compound-disease-target network. The pink triangle represents MIRI, the orange diamonds represent the active compounds of cinnamon, and the green circles represent the targets.

The active compounds and potential targets identified in the preceding steps were imported into Cytoscape to create the active compound-disease-target network diagram (Figure 2). Only the subnetwork with a high degree value was selected for visualization considering that it was impossible to depict the enormous number of targets. The analysis of the network revealed that the average degree of the 19 active compounds was 241.89, and oleic acid, stearic acid, palmitic acid, linoleic acid, beta-sitosterol, and eugenol were the top active compounds.

### 3.4. PPI Network Analysis

**Figure 3:**
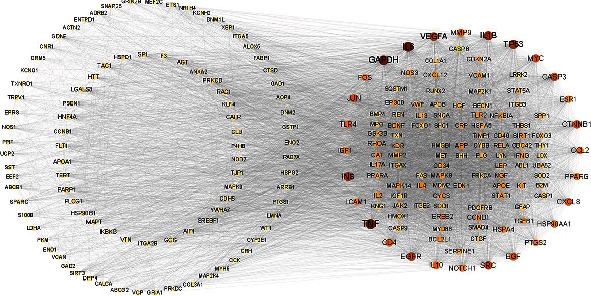
The PPI network. The shade of the color represents the magnitude of the target's degree. The greater the degree value, the darker the color.

To explore the mechanism underlying the therapeutic effects of cinnamon against MIRI, 1144 targets were imported to the STRING database to construct a PPI network. And the degree, closeness, and betweenness were calculated to be 51.64, 0.00038, and 1580.54, respectively. After screening according to these thresholds, we obtained 216 key targets (Supplementary Table S2) (Figure 3).

### 3.5. The Results of GO and KEGG Enrichment Analyses

**Figure 4:**
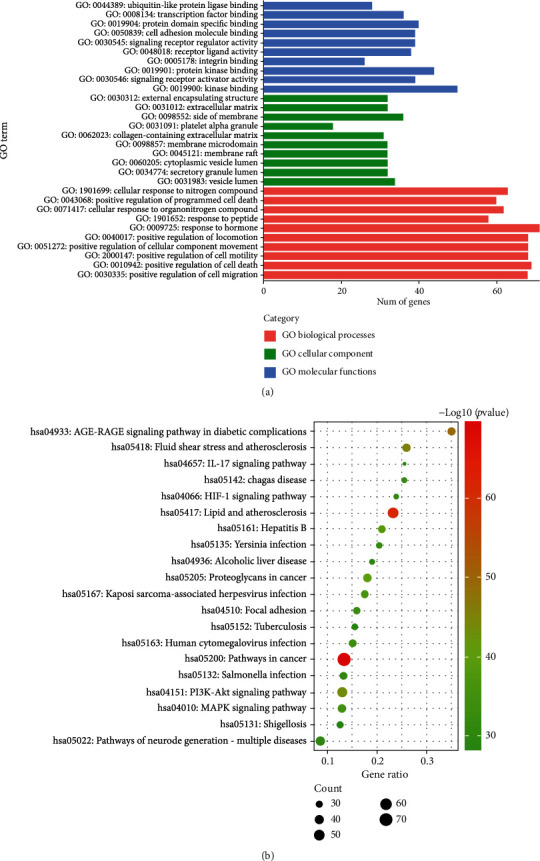
GO and KEGG enrichment analyses. (a) The top enrichment terms of BP, CC, and MF in the GO analysis. The *x*-axis represents the enrichment count of the target, and the *y*-axis represents the GO category of the target gene. (b) The top 20 significantly enriched pathways. The *x*-axis represents the enrichment score, and the *y*-axis represents the main pathway.

To determine the molecular mechanisms underlying cinnamon treatment of MIRI, we used Metascape to carry out GO biofunctional annotation and KEGG pathway enrichment analysis of the key targets. We obtained 3093 GO terms, which comprised 2675 biological process (BP) terms, 185 cellular component (CC) terms, and 233 molecular function (MF) terms. The top 10 considerably enriched terms for BP, CC, and MF are visualized in Figure 4(a). Results showed that the main BP terms were positive regulation of cell migration, cell death, cell motility, cellular component movement, and locomotion; the main CC terms were vesicle lumen, secretory granule lumen, cytoplasmic vesicle lumen, and membrane raft; the main MF terms were kinase binding, signaling receptor activator activity, protein kinase binding, and integrin binding.

We then identified 208 KEGG signaling pathways. The top 20 paths with the highest level of enrichment were chosen for visualization (Figure 4(b)). Results showed that the targets were enriched mainly in the lipid and atherosclerosis, PI3K-Akt, MAPK, and IL-17 signaling pathways. In addition to these signaling pathways, the HIF-1 signaling pathway was shown to be involved in the reprogramming of cellular energy metabolism, which suggested that cinnamon affects MIRI by interfering with HIF-1.

**Figure 5:**
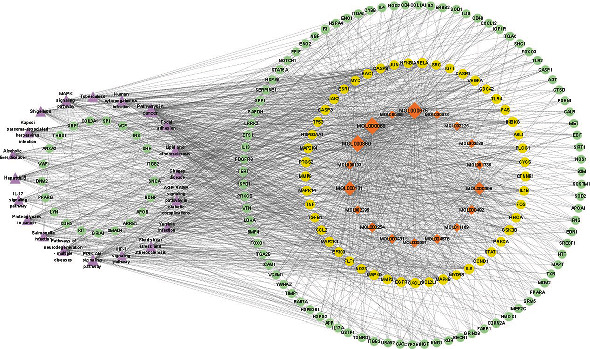
Compound-target-pathway network of cinnamon. The orange diamonds represent the active compounds of cinnamon. The green circles represent the targets with a low degree value. The yellow circles represent targets with a high degree value. The purple triangles represent pathways.

Furthermore, to better understand the mechanism of action of cinnamon in the treatment of MIRI, an active compound-target-pathway relationship network was built based on the enriched targets of the KEGG pathway (Figure 5). Results revealed an interaction between the active compound and the target as well as the related pathways of cinnamon for the treatment of MIRI.

### 3.6. Molecular Docking Results

**Figure 6:**
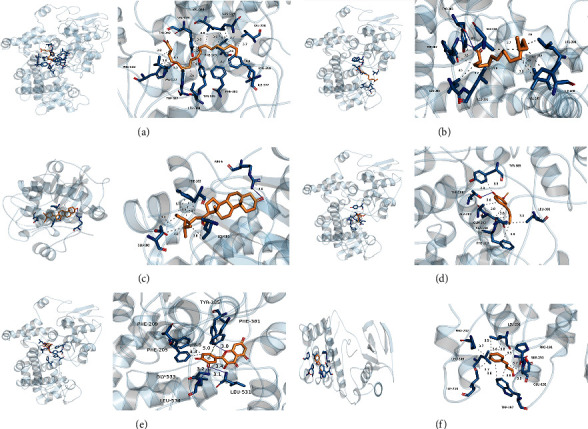
Molecular docking verification. (a) The docking mode of oleic acid and PTGS2. (b) The docking mode of palmitic acid and PTGS2. (c) The docking mode of beta-sitosterol and GSK3B. (d) The docking mode of eugenol and PTGS2. (e) The docking mode of taxifolin and PTGS2. (f) The docking mode of cinnamaldehyde and MAPK14.

To validate our findings, we used molecular docking to evaluate the interaction between the core active compounds and the targets. The binding affinity was less than −5.0 kcal/mol, which indicated a good interaction. Numerous significant targets, including prostaglandin-endoperoxide synthase 2 (PTGS2), glycogen synthase kinase 3 (GSK3B), and mitogen-activated protein kinase 14 (MAPK14), were docked to six primary active compounds in cinnamon. The binding affinity results are shown in Table 4. Oleic acid, palmitic acid, eugenol, and taxifolin have good binding affinity with PTGS2, beta-sitosterol has a good binding affinity with GSK3B, and cinnamaldehyde has a good binding affinity with MAP2K1. These results demonstrated that therapeutic benefit of the six compounds could have been achieved by PTGS2, GSK3B, and MAP2K1. The conformation of the core active compounds and targets is shown in Figure 6.

### 3.7. Hypoxia/Reoxygenation-Induced Cardiac Contractility Dysfunction

**Figure 7:**
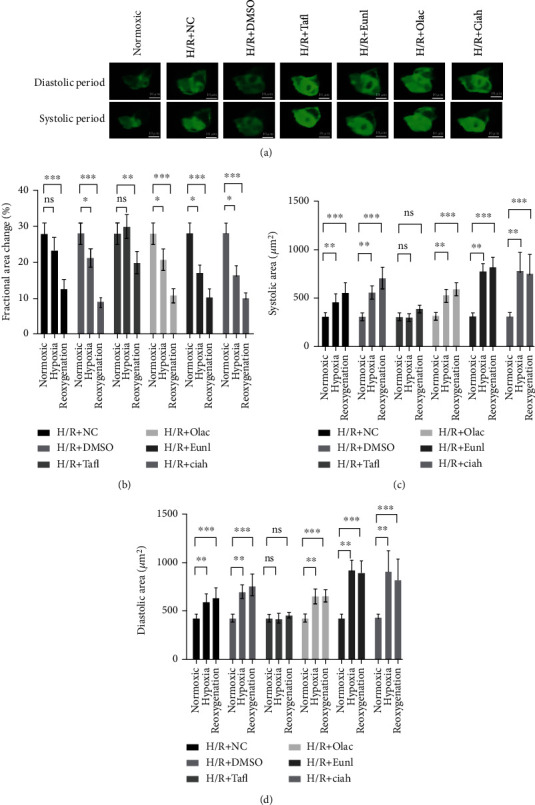
Taxifolin alleviated hypoxia/reoxygenation-induced cardiac dysfunction. Hypoxia/reoxygenation increased cardiac contractility dysfunction; however, it was reversed by taxifolin derived from cinnamon. Representative images of cardiac fluorescence imaging (a), quantification of FAC (b), SA (c), DA (d) in zebrafish larvae treated with hypoxia for 48 hours/reoxygenation for 2 hours (pretreated with PBS, 1% DMSO solvent, taxifolin, eugenol, oleic acid, and cinnamaldehyde, respectively) or normoxic water starting at 2 dpf. *N* = 7–9. ^∗^*P* < 0.05, ^∗∗^*P* < 0.01, ^∗∗∗^*P* < 0.001.

**Figure 8:**
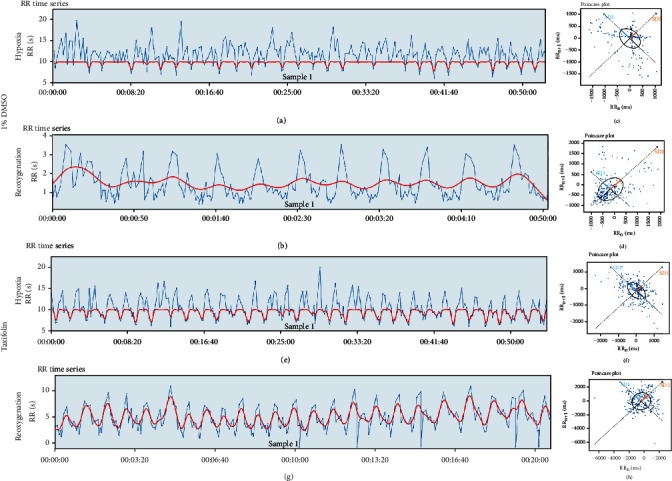
Taxifolin reduced arrhythmia risk after hypoxia-reoxygenation in zebrafish. Heart rate variability in zebrafish larvae. The tachocardiogram with RR intervals (the interval between two R waves, which means the heart beat cycle) in time (a, b, e, f). Nonlinear analysis and intrinsic cardiac properties (c, d, g, h). An increase in the duration of the RR intervals was observed on exposure to 1% DMSO (c, d), reflecting the occurrence of arrhythmia episodes. RRn and RRn + 1 are two consecutive intervals. However, protection from arrhythmias was observed when exposed to taxifolin (g, h).

To simulate the development of MIRI, embryos were transferred to a beaker of hypoxic water for 48 hours and subsequently normoxic water for 2 hours. Heart functions, including FAC, DA, and SA were measured using a digital high-speed camera. We observed a decrease in FAC and an increase in DA and SA, implying cardiac dysfunction and consequent functional compensation. We next evaluated the heart function of the zebrafish larvae after reoxygenation and observed the further deterioration after the recovery of oxygen, which indicated that the myocardium suffered from ischemia-reperfusion injury (Figures 7(a) and 7(b)). Notable, both hypoxia and hypoxia/reoxygenation increased the risk of arrhythmia (Figures 8(a)–8(d)).

### 3.8. Taxifolin from Cinnamon Alleviated Myocardial Ischemia-Reperfusion Injury

We screened four natural compounds from cinnamon including taxifolin, eugenol, oleic acid, and cinnamaldehyde and evaluated their potential effects on MIRI through the above network pharmacology analysis and previous reports. Two dpf, the zebrafish were initially treated with hypoxia for 48 hours and then transferred into normoxic water for 2 hours. Taxifolin, an active ingredient of cinnamon, has been shown to elicit anti-inflammatory and antioxidant stress pharmacological activity; however, its potential efficacy in MIRI prevention remains unknown. In the present study, we found that exposure to taxifolin attenuated hypoxia- and reoxygenation-induced loss of FAC and pathological cardiac compensation (Figures 7(a)–7(d)) in zebrafish and protects them from arrhythmias (Figures 8(e)–8(h)). Meanwhile, we failed to find the protective effects of eugenol, oleic acid and cinnamaldehyde on myocardial ischemia-reperfusion injury (Figures 7(a)–7(d)).

## 4. Discussion

Our study combining the DTI approach with network pharmacology revealed that the potential anti-MIRI compounds in cinnamon were oleic acid, palmitic acid, beta-sitosterol, eugenol, taxifolin, and cinnamaldehyde, which acted on important targets, including PTGS2, GSK3B, and MAPK14, as well as the signaling pathways of PI3K-Akt, MAPK, IL-7, and HIF-1.

Previous studies have demonstrated that oleic acid protects against cadmium-induced oxidative damage to the heart and liver tissues of male rats via the regulation of the antioxidant defense system, inflammatory response, and metabolic enzyme function [35]. Palmitic acid is a saturated fatty acid that plays a role in metabolic syndromes, cardiovascular diseases, cancer, neurodegenerative diseases, and inflammation by influencing molecular signaling [36–41]. Beta-sitosterol elicits anti-inflammatory [42], antioxidant [43], and immunomodulatory activity [44]. Eugenol is a phenolic aromatic molecule with antioxidant, anti-inflammatory, and antiapoptotic properties [45]. Eugenol has been studied as a therapy for ischemia-reperfusion damage. For example, eugenol protects against ischemia-reperfusion injury in heart transplant recipients by reducing the inflammatory response, apoptosis [46]. Taxifolin is involved in a variety of pharmacological processes, including antioxidant processes, mitochondrial protection, and advanced glycation end-product formation and suppression [47]. It has become increasingly valuable in the treatment of cancer, cardiovascular diseases, and chronic hepatitis, among other diseases [48]. Cinnamaldehyde is an aldehyde organic compound with anti-inflammatory, antioxidant, and vascular protective properties [49, 50] and has shown considerable promise in the prevention and treatment of cardiovascular diseases. Taken together, these studies demonstrate the potential of these compounds in the treatment of MIRI.

Furthermore, we built a PPI network and obtained the top 10 target proteins of cinnamon against MIRI-mediated cardiac dysfunction (TNF, IL6, GAPDH, IL1B, TP53, INS, VEGFA, EGFR, SRC, and CTNNB1) by degree value. According to the core target proteins, the GO enrichment analysis demonstrated that target proteins of cinnamon core components are mainly involved in the positive regulation of cell death or migration, and these cellular biological behaviors play a crucial role in aggravating MIRI and subsequent cardiac dysfunction. In addition, KEGG enrichment analysis showed that cinnamon could interfere with a variety of MIRI signal pathways, especially the PI3K-Akt, MAPK, and IL-17 signaling pathways, which are involved in inflammation and oxidative stress. The PI3K-Akt signaling pathway has been detected in numerous different types of cells and is involved in the physiological processes of various diseases, including cancer, myocardial infarction, and heart failure. Icariside II is a bioflavonoid compound that has a positive effect on MIRI by activating the PI3K-Akt signaling pathway and inhibiting inflammation and cardiomyocyte apoptosis [51]. The MAPK signaling pathway, which is involved in a cascade of multilevel protein kinases, such as JNK and p38 MAPK, has been implicated in apoptosis and inflammation [52]. By silencing integrin *β*3 (ITGB3), the MAPK signaling pathway becomes activated, which increases the phosphorylation of downstream GSK-3 and Cx43, promotes mouse cardiomyocyte proliferation, inhibits apoptosis and the inflammatory response in cardiomyocytes, and provides protection against MIRI [53]. In turn, the IL-7 signaling pathway is important for a variety of inflammatory responses [54]. Macrophages play an important role in MIRI development, and IL-7 can improve MIRI by promoting cardiomyocyte apoptosis via macrophage infiltration and polarization [55]. Surprisingly, the HIF-1 signaling pathway was discovered to be linked to cellular energy metabolism reprogramming. HIF-1 is a transcription factor that comprises two subunits: HIF-1*α* and HIF-1*β*. HIF-1 is an oxygen-sensitive transcription factor that drives the adaptive metabolic response to hypoxia and is important in MIRI [56]. Through the HIF-1/BNIP3 pathway, berberine is thought to enhance mitochondrial autophagy, lower cardiac enzyme activity, induce cardiomyocyte proliferation, block cardiomyocyte apoptosis, and protect the heart against MIRI [57].

PTGS2 is a subtype of prostaglandin-endoperoxide synthase and plays a specific role in the inflammatory response. It has been demonstrated that inhibiting PTGS2 activates the MAPK pathway, thereby reducing the inflammatory response and improving myocardial remodeling in mice with myocardial infarction [58]. GSK3B plays a significant role in the treatment of MIRI and is involved in energy metabolism, inflammation, apoptosis, and the oxidative stress response [59]. MAPK14, also known as p38a, is involved in cell proliferation, apoptosis, and the inflammatory response. An increasing number of studies have highlighted the significance of MAPK14 in the treatment of myocardial inflammatory diseases [60, 61]. Therefore, PTGS2, GSK3B, and MAPK14 may serve as target proteins related to MIRI.

Therefore, we conducted animal experiments to validate the active compounds. Due to the limitations of funding and experimental equipment, only four compounds were selected for verification. Fortunately, the potential protective effect of taxifolin on MIRI was observed in the zebrafish experiment. Notably, the solvent DMSO used in our experiments had nonnegligible zebrafish cardiotoxicity, which may lead to an underestimation of the MIRI-protective effects of other main components of cinnamon and underscore the need for process optimization for the extraction and preservation of active ingredients in Chinese herbal medicines. Nonetheless, considering that zebrafish shares high homology to human MIRI pathology mechanisms, our experimental results may provide new insights for further research.

The network pharmacology approach proposed in this study may achieve promising prediction results in terms of active compounds, corresponding targets, and pathways. However, because of the inherent nature of network pharmacology, it was not possible to obtain all compounds and targets via databases and analysis software. Moreover, even if the DTI model was used to expand the number of targets, the problem could not be entirely solved. Furthermore, relevant data could not be used to train the model to improve performance because of the difficulty in obtaining negative samples. Therefore, there is still room for improvement in future work in terms of the quality and quantity of training data.

## 5. Conclusion

In this study, we proposed a deep learning-based network pharmacology approach. With the aid of the DTI method, we predicted six potential active compounds and three targets, and their validity was assessed using molecular docking and an animal experiment. Although only one active compound, taxifolin, was finally demonstrated to have a protective effect against MIRI by the zebrafish model, we believe that more promising findings can be obtained if further experiments are conducted. Anyway, the aforementioned analysis of the prediction compounds, targets, and pathways according to other studies suggested that cinnamon may be considered as a potential for effective treatment of MIRI.

## Figures and Tables

**Table 1:** Basic information about the active compounds of cinnamon.

MOL ID	Compound	OB (%)	DL
MOL001736	(−)-taxifolin	60.51	0.27
MOL000358	Beta-sitosterol	36.91	0.75
MOL000359	Sitosterol	36.91	0.75
MOL000492	(+)-catechin	54.83	0.24
MOL000073	Ent-Epicatechin	48.96	0.24
MOL004576	Taxifolin	57.84	0.27
MOL011169	Peroxyergosterol	44.39	0.82
MOL002295	Cinnamic acid	19.68	0.03
MOL000991	Cinnamaldehyde	31.99	0.02
MOL000254	Eugenol	56.24	0.04
MOL000103	PHB	30.15	0.03
MOL000431	Coumarin	29.17	0.04
MOL000131	Linoleic acid	41.9	0.14
MOL000675	Oleic acid	33.13	0.14
MOL000860	Stearic acid	17.83	0.14
MOL000069	Palmitic acid	19.3	0.1
MOL002046	Caproic acid	73.08	0.01
MOL003530	Methoxycinnamaldehyde	26.52	0.04
MOL002225	Cinnamyl alcohol	38.35	0.02

**Table 2:** Metric results of HyperAttentionDTI.

Fold	Accuracy	Precision	Recall	AUC
1	0.87175	0.77303	0.78806	0.93310
2	0.87776	0.79910	0.74773	0.93108
3	0.86787	0.75450	0.78324	0.91642
4	0.87427	0.79713	0.75288	0.92480
5	0.87490	0.79213	0.76371	0.92186

**Table 3:** The number of targets predicted for each compound by HyperAttentionDTI.

MOL ID	Name	Number
MOL000431	Coumarin	52
MOL000069	Palmitic acid	908
MOL000254	Eugenol	148
MOL000860	Stearic acid	908
MOL000492	(+)-catechin	95
MOL000358	Beta-sitosterol	477
MOL004576	Taxifolin	31
MOL002295	Cinnamic acid	27
MOL000675	Oleic acid	886
MOL000991	Cinnamaldehyde	28
MOL001736	(−)-taxifolin	31
MOL000131	Linoleic acid	623

**Table 4:** Molecular docking results for the active compounds of cinnamon.

Compound	Target	PDB ID	Score
Oleic acid	CXCL8	4xdx	−6.1
	PTGS2	5f19	−7.5
	JAK2	7f7w	−5.9

Palmitic acid	PTGS2	5f19	−5.7
	CXCL8	4xdx	−5.2
	GSK3B	1o6l	−5.2

Beta-sitosterol	GSK3B	1o6l	−9.9
	JAK2	7f7w	−9.1
	MAP2K1	3eqc	−8.7

Eugenol	GSK3B	1o6l	−6.3
	PTGS2	5f19	−6.6
	JAK2	7f7w	−6.5

Taxifolin	PTGS2	5f19	−9.9
	JAK2	7f7w	−9.1
	MAP2K1	3eqc	−8.8

Cinnamaldehyde	MAPK14	3lff	−6.4
	PTGS2	5f19	−6.3
	JAK2	7f7w	−6.1

## Data Availability

The datasets analyzed during the current study are available in the https://github.com/2020-xuetao/MIRI.

## Supplementary Materials

Supplementary Materials Table S1: the intersection targets. Table S2: the data of topological parameters of the PPI network. (Supplementary Materials).
