# Supplementary material for: Exploring Myocardial Ischemia-Reperfusion Injury Mechanism of Cinnamon by Network Pharmacology, Molecular Docking, and Experiment Validation
Source: Comput Math Methods Med. 2023 Feb 23;2023:1066057. doi: 10.1155/2023/1066057 (PMC9981296; doi:10.1155/2023/1066057)
Supplement: Supplementary Materials — Table S1: the intersection targets. Table S2: the data of topological parameters of the PPI network. (Supplementary Materials). [file 1066057.f1.pdf]

# Supporting Information

## Exploring Myocardial Ischemia-Reperfusion Injury Mechanism of Cinnamon by Network Pharmacology, Molecular Docking, and Experiment Validation


**Authors:**

Tao Xue[1+], Yan Xue[2, 3, 4+], Yangyue Fang[1], Chuanghong Lu[2, 3, 4], Yu Fu[1], Zefeng Lai[6], Xiaojun Qin[6], Feng Huang[2, 3, 4*], Zhiyu Zeng[3, 4, 5*] and Jianping Huang[1*]

**Affiliations:**

[1] Alibaba Business School, Hangzhou Normal University, Hangzhou 310000, China
[2] Department of Cardiology, The First Affiliated Hospital of Guangxi Medical University, Nanning 53000, China
[3] Guangxi Key Laboratory of Precision Medicine in Cardio-Cerebrovascular Diseases Control and Prevention, Nanning 53000, China
[4] Guangxi Clinical Research Center for Cardio-Cerebrovascular Diseases, Nanning 53000, China
[5] Department of Geriatric Cardiology, The First Affiliated Hospital of Guangxi Medical University, Nanning 53000, China
[6] Pharmaceutical College, Guangxi Medical University, Nanning 53000, China

**Corresponding author:**

Feng Huang, MD
Zhiyu Zeng, MD
Jianping Huang, Ph.D

**Email:**

huangfeng3000@126.com
zengzhiyu@gxmu.edu.cn
hjp@hznu.edu.cn



**Acknowledgements**

This work was supported by Guangxi Key Laboratory of Precision Medicine in Cardio-Cerebrovascular Diseases Control and Prevention (No. 19-245-34), Guangxi Clinical Research Center for Cardio-Cerebrovascular Diseases (No. AD17129014), the National Natural Science Foundation of China (No. 82070279, 81960082, 81760057, 61873080), and the Natural Science Foundation of Guangxi Province (No. 2016GXNSFAA380002, 2018GXNSFAA050059).

**Table S1 The Intersection Targets**

| GENE NAME | | | | | |
|---|---|---|---|---|---|
| MBL2 | GP1BA | SLC25A4 | APOA1 | CDON | ADAM17 |
| MME | JAK2 | MYC | CAT | PIAS1 | HMCN1 |
| GAPDH | GSK3B | EPOR | MMP2 | SMAD1 | YAP1 |
| F2R | LAMA4 | XIAP | GCLC | COL6A1 | ENO1 |
| SLC9A3R2 | VCAN | FOXP2 | LEP | S100P | SENP3 |
| BMP6 | TEK | SOCS3 | ENO2 | COL6A5 | MAOB |
| PDE5A | SIRT3 | CHI3L1 | NFE2L2 | NID2 | RHOB |
| CIITA | IRAK1 | ABCC8 | KCNJ5 | ANK2 | TRPM7 |
| EPRS1 | PRKCE | IL37 | HGF | KLRK1 | FHL2 |
| P2RY12 | MERTK | ZC3H12A | ABCC9 | PDGFA | TGFBI |
| NOD2 | EPHA3 | FLNA | RYR2 | CMKLR1 | TOLLIP |
| IL1R1 | ABL1 | RHOA | ADM | VSIG4 | ITGA7 |
| STAT1 | MAP2K3 | VTN | CD14 | SNAP25 | ALOX15 |
| NFKB1 | TNNI3K | ADCYAP1 | AGER | AASS | TJP1 |
| CALR | ROCK1 | MMP14 | NOS1 | BAD | HADHB |
| CTF1 | LRP2 | UMOD | ABCA1 | VEGFC | WT1 |
| UTS2 | MGAM | CASP7 | TIMP1 | ADAMTS5 | GRIK1 |
| SCARB1 | FRAS1 | IL11 | REN | P4HB | TRPC6 |
| ANGPT2 | ATR | TSPO | PARP1 | RHOD | COL6A3 |
| CD59 | AXL | CTSD | MYH6 | MAP3K14 | CYP2B6 |
| THBS2 | MAP2K4 | CXCR3 | CLU | PRELP | KCNMA1 |
| ADORA1 | TYK2 | NAMPT | PRKAG2 | TINAGL1 | AREG |
| APP | ROR1 | ENTPD1 | ITGA2B | SPON1 | SHC1 |
| VIP | GRIN2B | NDUFS4 | LGALS3 | ADAMTS20 | DIAPH1 |
| C1S | CDK5 | AMPD1 | CD40LG | COL20A1 | EIF2AK3 |
| CDKN1A | ENG | PRKCD | APLN | CHADL | CFP |
| EGR1 | PRKDC | KLK1 | CALCA | SSPOP | IDO1 |
| ITGAL | ADAM12 | IL3 | IL2 | ERCC2 | GPR55 |
| AIF1 | SLK | SETD2 | GHRL | ZFYVE9 | STAT5B |
| ASIC1 | EGFR | APLNR | CYP2C9 | ATP2B4 | CD80 |
| TNFRSF1B | GSDME | TGM2 | HSPG2 | APAF1 | FGF4 |
| PDGFB | CRP | DUSP1 | PROCR | RBM28 | ANGPTL4 |
| RAC1 | MPO | SMAD4 | PLA2G7 | AIMP1 | GADD45A |
| S100A9 | LTA | ACP1 | CDKN2A | NDUFB2 | CCK |
| GSTM1 | NOS3 | CASP2 | FLT1 | GRIA1 | LAMA1 |
| THPO | VWF | VCP | CDH5 | GHRH | SEMA7A |
| BCL2L1 | CXCL8 | CAST | CP | DUSP6 | STAT5A |
| NTN1 | HMOX1 | BAK1 | PTGIS | TOMM40 | BCL2L15 |
| TNNI3 | NOS2 | ALOX12 | CD40 | TNFRSF10A | BLVRB |
| SDC1 | SELP | IL22 | CPT2 | BIRC2 | CPOX |
| ABRAXAS2 | TP53 | RNF182 | HSPB1 | FOXC2 | P2RY11 |
| C5AR1 | LCN2 | NLRP1 | SMARCA4 | PPP1R15A | AOC1 |

| | | | | | |
|---|---|---|---|---|---|
| DNM1L | IL18 | TNFSF12 | CD34 | CPT1B | DUSP19 |
| MAX | SCG2 | XRCC1 | SENP1 | KCNT2 | TFF3 |
| EEA1 | ANGPTL2 | TFAM | PSMD9 | P2RY14 | BCL2A1 |
| CD4 | FGF19 | TLR3 | ITGAX | MUC20 | CCR3 |
| SERPINA5 | PTGER2 | MAPK8IP3 | PRSS2 | CHD8 | PKD1 |
| LHFPL3 | CTTN | ADGRL1 | MYBPC1 | MAT1A | MYO1E |
| RABEP1 | FPR2 | ERCC1 | SMPD2 | F12 | INSIG1 |
| FOXP1 | MIEF2 | EP300 | GSS | CTHRC1 | DDOST |
| SNAP29 | OGG1 | GRIK5 | EIF4G1 | CD300LB | LPAR1 |
| VTI1B | LAIR1 | GNAI3 | PLCG1 | ANKRD23 | AP4M1 |
| NEO1 | DNM2 | ADCYAP1R1 | SLC29A4 | CCDC88B | SORT1 |
| IRAG2 | TNFSF9 | EDN2 | KCNQ4 | CLEC9A | PDK2 |
| VAMP8 | PDIA2 | CYP2E1 | AKAP13 | MEGF10 | FOXO3 |
| NAPA | RAMP1 | ABCC5 | ITGB3 | TET2 | ADGRB1 |
| KCNH1 | TRIM28 | CASP12 | B2M | HTT | NPHS1 |
| SPAM1 | OGDH | PPP3CA | GRIN2A | DPPA2 | DYSF |
| CACNA1H | CA1 | POLB | IL6R | WTIP | CAVIN1 |
| VAMP3 | CELSR1 | PKLR | SMARCAL1 | USP53 | TP63 |
| OTOF | AGFG1 | CHRNG | MEF2C | SRXN1 | LITAF |
| AKAP9 | ATG12 | ACTN2 | SERPINA3 | LGALSL | CCS |
| RIMS1 | MLKL | ST6GAL1 | SST | INAVA | GRM5 |
| VAV3 | SLC5A2 | CENPE | SLC12A2 | NECAB2 | BHLHE40 |
| OPA1 | JMJD1C | DAG1 | F11 | TMEM119 | SCN10A |
| CAV2 | RRAD | ERCC5 | EDNRA | THBS1 | CBLB |
| MYH9 | MSRA | GLP1R | PAPPA | ABCB1 | ADAMTS7 |
| KLK6 | PEPD | GRN | SIAH2 | TERT | RARRES1 |
| KCNB1 | GPER1 | ICAM3 | TOMM20 | SULT1A3 | TLR9 |
| KCNB2 | ATP5F1B | IGFBP1 | CA3 | GPX1 | GP6 |
| KCNC3 | MCUB | IL4R | KCNN1 | HSP90B1 | FSTL1 |
| KCNE2 | BCAR1 | BCR | ADAMTSL1 | MYH10 | SPTLC1 |
| KCNG2 | KCNN3 | MITF | NFAT5 | LONP1 | STIM1 |
| KCNG4 | NCK1 | PTN | MAPK8IP1 | ATP1A1 | UBR1 |
| KCNH4 | GSTM3 | OMG | ACACA | KCNH2 | SIGLEC10 |
| KCNH6 | NFE2L1 | SKI | GCH1 | MAP2 | SLC2A9 |
| KCNQ3 | GDNF | BMI1 | CABIN1 | VIPR1 | MAGI2 |
| KCNQ5 | RETN | CCN3 | MEF2A | MYL4 | OLIG1 |
| THY1 | TRAF2 | PIM1 | MFF | RLF | TRPM4 |
| ETS1 | CD47 | PKM | C1D | USP6 | SMOC2 |
| VEGFD | ABCB11 | SREBF1 | ADAMTS1 | KCNC4 | SLC17A8 |
| SOCS1 | TRPM2 | ZEB1 | CADPS | TXNRD2 | TRAK1 |
| EIF2S1 | ABCB4 | ITGAV | SLC16A1 | DEK | STOML3 |
| CCNB1 | MPL | YIPF6 | NPY1R | JAG1 | MMP28 |
| CCL11 | GREM1 | FOXA2 | SLC4A4 | SCN11A | AHI1 |
| ZNF354A | TRAF7 | BDKRB1 | ARF4 | LGR5 | CYP27B1 |

| | | | | | |
|---|---|---|---|---|---|
| VEGFA | SARM1 | SIRT1 | CUL3 | APOL4 | HMOX2 |
| TSC2 | ANKRD1 | FAS | TACR1 | EMILIN3 | ALDH9A1 |
| C4B | SRC | CCL3 | MLC1 | DDT | CD69 |
| EPAS1 | NPR1 | HSPA4 | RIPK3 | CRHR2 | PROC |
| MASP2 | MAPK9 | MAPT | ADAMTS3 | GLRX | NES |
| CLCN3 | RIPK2 | ADRB1 | ATF6 | GCKR | IL23A |
| IKBKG | TIE1 | PLA2G6 | IFIT3 | TLR7 | GAD1 |
| ATP2A1 | KIT | SELPLG | FPR1 | PPP1CA | ABCG2 |
| ADK | ANO1 | LRP6 | SMPD1 | PNPLA8 | AHSG |
| ERBB2 | MAPK11 | ACE2 | EGLN3 | DDIT4 | VEGFB |
| LYN | RAF1 | CCR5 | YBX1 | METTL14 | NLRX1 |
| ATP7A | RYK | EGF | RBP3 | AKIP1 | IL18BP |
| CCR1 | DSG1 | SH2B3 | TRPC5 | SIVA1 | GPX3 |
| TRPM8 | DCC | HSPD1 | DGKQ | SP3 | BAG1 |
| IL6 | MET | CRYAB | KL | F11R | FOXC1 |
| TNF | YES1 | CASP9 | TRAF5 | SNHG12 | ATF4 |
| SERPINE1 | DDR1 | SDHB | ODC1 | CD58 | GLO1 |
| IL10 | MAPK10 | NEFL | YRDC | GORASP1 | CXCL6 |
| SOD1 | GFAP | IL13 | EEF2 | KCNJ1 | SPARC |
| MMP9 | MAPK14 | TKT | LNP1 | YWHAZ | BGN |
| ESR1 | CXCL1 | CSF1 | KCNF1 | CYGB | COL14A1 |
| CCL2 | MAP2K1 | HSPA5 | KCNH5 | PSMA2 | NDRG4 |
| S100B | LTF | S100A8 | IL6ST | AQP8 | GRP |
| EDN1 | HSPB2 | IL5 | TXNRD1 | USP18 | TNFRSF12A |
| F3 | SIRPA | KCNQ1 | SLC6A11 | MSRB1 | BMP4 |
| EPO | CSK | COL7A1 | ADGRL3 | IL18RAP | PPIF |
| F5 | PTPA | BECN1 | SLCO1B3 | JMJD6 | E2F1 |
| LMNA | PDGFRB | FGF7 | GNAI2 | NDUFA4L2 | GNAI1 |
| IGF1 | ANG | S1PR1 | CLDN4 | STK4 | LAMB2 |
| KNG1 | TTK | VHL | MT-CYB | TRAF1 | SRF |
| CXCL12 | THBD | PSEN1 | PCSK2 | FKBP4 | RPS6KB1 |
| XDH | OLR1 | PSAP | APOE | BFAR | GJC1 |
| VCAM1 | ADAMTS13 | GZMB | IDH2 | EIF4EBP1 | MCAM |
| FGB | CTNNB1 | CTSG | PAX2 | SLC51B | CTSS |
| CCN2 | TLR2 | SORD | CEBPB | CCR2 | APOM |
| F10 | PPARA | TRPV4 | P2RX4 | CYP2C19 | HMGB2 |
| AGT | AQP4 | CRH | SEMA3A | PLA2G2A | ATF2 |
| PLG | GLS | KCNJ8 | SLC22A4 | SERPINF2 | CLEC4A |
| ADRB2 | CYBB | UCN | PTER | MMP13 | TFG |
| SOD2 | DPP4 | ACHE | MICU1 | LDLR | CTH |
| ALDH2 | ADORA3 | HTRA2 | GPR180 | CD163 | UTS2B |
| SPP1 | CYSLTR2 | C3AR1 | WFIKKN1 | PCSK9 | CCL22 |
| CCL5 | NFKBIA | PTGDS | SLC6A4 | PNP | ADAMTS8 |
| RSPO1 | FCN3 | SLC9A1 | ASCL1 | HDAC3 | BNIP3 |

| | | | | | |
|---|---|---|---|---|---|
| REG4 | NUCB2 | HSPA1A | CACNA2D1 | SLC1A1 | MDM2 |
| WFIKKN2 | MARCHF5 | CCND1 | CEBPD | ARRB1 | LGALS1 |
| EPC1 | FAM3A | FOS | GAD2 | ATP5F1E | RUNX1 |
| PLA2G3 | TMEM123 | IRF5 | GLRA1 | MAP3K8 | TXN2 |
| APIP | LTC4S | DCN | SERPIND1 | SERPING1 | SERPINF1 |
| SPEG | GIP | FASLG | ITGB6 | BEST1 | NOD1 |
| FOXK1 | POMZP3 | MCU | JUND | NR4A2 | RUNX2 |
| MXRA8 | USO1 | TF | PDE1A | GPR31 | DLD |
| OLFM2 | UBA52 | TIMP2 | PLA2G1B | PF4 | IL9 |
| CREG1 | LHFPL6 | AHSP | MMP7 | BDKRB2 | SIRT6 |
| PACC1 | UFD1 | PLAU | CD6 | SERPINI1 | CLEC3B |
| HPCAL4 | RAB8A | BMP7 | HNF1B | CTSL | SLC22A3 |
| VNN1 | NAPG | NPPC | NOTCH1 | BDH1 | S1PR3 |
| CCL21 | ROPN1B | CX3CR1 | TBL1X | PRNP | TIMP4 |
| CD93 | DOC2B | MYD88 | PTGIR | APOC3 | TMSB4X |
| NPPB | SNAP23 | FABP1 | HNF4A | MYDGF | TGIF1 |
| IL1B | VPS33B | TNNC1 | GAS6 | ACADS | FEM1A |
| NPPA | NOX5 | FGF1 | VSNL1 | FXYD1 | ADAM10 |
| TGFB1 | ADAM9 | GSTP1 | P2RX2 | MYL3 | PTHLH |
| HMGB1 | NMNAT3 | ADM2 | FAT1 | PCSK1 | P2RY1 |
| IFNG | KCNC1 | LOX | NACA | IZUMO1 | GLRX2 |
| GCLM | KCNC2 | IL15 | SGCD | MYMX | ATG5 |
| NGF | KCND1 | RELA | SQSTM1 | FAU | COL15A1 |
| IL4 | KCND2 | IL7 | PROX1 | MAP1B | C1R |
| CYCS | KCND3 | FOXO1 | RCAN1 | LHFPL1 | IL7R |
| HAVCR1 | KCNE4 | LAMA2 | PSPN | MFN1 | PLA2G10 |
| HTR2A | KCNG3 | ELAVL1 | LRRFIP1 | STX6 | THAP5 |
| TXN | KCNS1 | FCGR3B | KCNK6 | NPLOC4 | GSTA2 |
| CBS | KCNAB1 | DDIT3 | SYT7 | BNIP1 | PPP1CC |
| CD46 | ITGA5 | GJB1 | PLSCR1 | VAMP7 | IMMT |
| ANGPT1 | INPP5D | SNCA | LBX1 | PRDX6 | H2AX |
| COL3A1 | EIF2A | TFPI | AIM2 | MMP12 | ADCY1 |
| CASP1 | PRSS1 | GAST | SRP54 | PRKCQ | HSPA1B |
| IL1RN | TMBIM6 | FBXO32 | ABCC4 | KCNE3 | AOX1 |
| BAX | TBXAS1 | XBP1 | IL27RA | EFNB2 | LAP3 |
| ITGB2 | HRH3 | OSM | PNKP | ID3 | HEXIM1 |
| CHAT | CDKN1B | IGFBP7 | CHRNA10 | CCNG1 | PSMA7 |
| GATA4 | CYP19A1 | PPIA | OLIG2 | STC1 | HTR2B |
| MIF | CYP3A5 | MUC1 | HOPX | STAT6 | PLSCR3 |
| IL17A | MT-ATP6 | LRG1 | BHLHA15 | BTG2 | RGS16 |
| CXCL10 | ARG1 | ANXA2 | NMNAT1 | CCNE1 | LDHA |
| CXCL2 | HLA-B | SLC24A3 | LRRK2 | FOSL1 | DEFB4A |
| CASP6 | GCG | KLF4 | NR3C2 | ID1 | MMP1 |
| SCFD1 | CDC42 | RAB7A | BCHE | LGALS7 | IFNB1 |

| | | | | | |
|---|---|---|---|---|---|
| FOSL2 | TAC1 | CASP4 | AKR1B1 | NFIB | HDAC6 |
| CD2AP | S100A6 | FLNB | ICAM1 | P2RX1 | SLC6A3 |
| MT-ATP8 | BLOC1S1 | LHX2 | APOB | HMMR | EGLN1 |
| MMP3 | TNNI1 | NR1H4 | CNR1 | SOX15 | CXCR2 |
| OXT | HCRT | BVES | CNR2 | CCL13 | KDR |
| CEL | SETMAR | RTN4R | NR1H2 | PITX2 | ERN1 |
| C1QA | AKT1S1 | SCGB3A2 | SHH | KLF6 | FABP4 |
| EPHX1 | KCTD9 | DEFB103A | ADORA2A | CASP3 | PPARG |
| FGFR4 | PTGS2 | GPR78 | TRPV1 | CASP8 | FABP3 |
| FGFR1 | HSP90AA1 | ACMSD | MAPK8 | PRKCA | PPARD |
| CD74 | CHRM3 | PELI3 | FASN | PON1 | PTGES |
| PTPRZ1 | SCN5A | SPINK6 | MAOA | NR1H3 | LPL |
| KDM6A | PDE3A | GFRAL | TLR4 | HMGCR | BDNF |
| CRLF2 | OPRM1 | PTGS1 | TRPA1 | G6PD | INS |
| CD24 | BCL2 | ADORA2B | ALOX5 | SLC6A2 | UCP2 |
| SP1 | JUN | FABP2 | HSD11B1 | GPBAR1 | |
| COL1A1 | AOC3 | IGF1R | NR4A1 | SOAT1 | |

**Table S2 he Data of Topological Parameters of The PPI Network**

| GENE | BETWEENNESS | CLOSENESS | DEGREE |
|---|---|---|---|
| MYC | 22237.2381 | 0.00049554 | 297 |
| SIRT1 | 9664.312401 | 0.000457875 | 177 |
| MPO | 1799.235328 | 0.000434783 | 121 |
| CCL2 | 5732.332608 | 0.000474834 | 245 |
| GAPDH | 52889.71325 | 0.000537346 | 431 |
| APOB | 3962.648919 | 0.000428449 | 106 |
| APOA1 | 2892.439967 | 0.000424268 | 99 |
| APOE | 9514.033309 | 0.000461681 | 183 |
| CRP | 8501.621437 | 0.000447227 | 163 |
| TLR2 | 5336.009959 | 0.000456621 | 197 |
| EP300 | 9917.500861 | 0.000460405 | 180 |
| IL1B | 27226.54647 | 0.000517331 | 378 |
| ICAM1 | 5202.954022 | 0.000467946 | 220 |
| APP | 15283.91069 | 0.00046729 | 195 |
| PPARG | 12383.94306 | 0.000475964 | 223 |
| VCAM1 | 2631.262192 | 0.000449843 | 184 |
| FABP1 | 1881.6776 | 0.000399521 | 57 |
| NOS3 | 10284.91743 | 0.000464037 | 177 |
| LEP | 8925.782178 | 0.00044964 | 168 |
| CLU | 4143.231266 | 0.000424088 | 85 |
| PSEN1 | 1875.489497 | 0.000429185 | 86 |
| SNCA | 9488.568834 | 0.000440141 | 117 |
| SREBF1 | 2104.897976 | 0.000423908 | 93 |
| MMP9 | 9883.998677 | 0.000480769 | 269 |
| TLR4 | 12858.57066 | 0.000484262 | 262 |
| RHOA | 10991.58208 | 0.000465983 | 190 |
| IL10 | 9700.843882 | 0.000481464 | 267 |
| IL6 | 40409.98725 | 0.000535332 | 439 |
| INS | 40930.74841 | 0.000520021 | 362 |
| TNF | 48101.48299 | 0.000542299 | 452 |
| NR1H4 | 2151.22775 | 0.00040833 | 56 |
| CDC42 | 8783.968905 | 0.000455166 | 155 |
| PPARA | 10079.85996 | 0.000449843 | 155 |
| ABCB1 | 4207.177985 | 0.000417537 | 66 |
| CD4 | 16410.77617 | 0.000484027 | 273 |
| CCND1 | 4820.392409 | 0.000463177 | 197 |
| ABCG2 | 4593.496555 | 0.000406504 | 56 |
| KDR | 6530.344001 | 0.000453309 | 165 |
| EGF | 16574.57967 | 0.000490436 | 286 |
| TP53 | 46966.79921 | 0.000518135 | 371 |
| ERBB2 | 5761.224451 | 0.000462535 | 197 |
| EGFR | 31394.37456 | 0.000507357 | 335 |

| | | | |
|---|---|---|---|
| NOTCH1 | 7675.154896 | 0.000464468 | 207 |
| TJP1 | 9814.728118 | 0.000435161 | 100 |
| KIT | 3124.643237 | 0.000445434 | 155 |
| BCL2L1 | 6997.187399 | 0.000453515 | 174 |
| HSPA4 | 11867.49178 | 0.000468823 | 199 |
| CD34 | 3575.865073 | 0.000447227 | 171 |
| CASP3 | 19455.22329 | 0.000498504 | 306 |
| HNF4A | 3044.871616 | 0.000426985 | 96 |
| CASP9 | 2097.866561 | 0.000437254 | 126 |
| HSP90AA1 | 17724.09336 | 0.000479846 | 235 |
| CTNNB1 | 28195.3039 | 0.000500501 | 309 |
| ANXA2 | 2599.328727 | 0.000434594 | 87 |
| HTT | 2924.369091 | 0.000429369 | 93 |
| PTGS2 | 9709.697531 | 0.000463822 | 205 |
| JUN | 14117.58936 | 0.000492611 | 294 |
| ABL1 | 2166.277303 | 0.000432152 | 108 |
| SRC | 36928.74378 | 0.000506586 | 323 |
| MAPK8 | 3338.84293 | 0.000453515 | 170 |
| GSTP1 | 2142.096088 | 0.000396668 | 57 |
| RELA | 3940.492539 | 0.000446429 | 155 |
| ESR1 | 10017.009 | 0.000470146 | 211 |
| CYP2E1 | 1710.609468 | 0.000406504 | 61 |
| VEGFA | 23399.44977 | 0.000506329 | 343 |
| LGALS3 | 2998.822667 | 0.000427716 | 97 |
| DPP4 | 4585.751093 | 0.000424088 | 75 |
| KCNQ1 | 4937.5902 | 0.000378072 | 56 |
| SNAP25 | 7526.995908 | 0.000401768 | 71 |
| KCNH2 | 5623.82275 | 0.000380084 | 61 |
| SST | 2253.625851 | 0.000418235 | 68 |
| CNR1 | 1916.953369 | 0.000416667 | 65 |
| GAD2 | 2997.595789 | 0.000402901 | 55 |
| UCP2 | 1907.212265 | 0.000406835 | 52 |
| GCG | 4205.995888 | 0.00042517 | 93 |
| AQP4 | 3582.736885 | 0.000412031 | 54 |
| ACTN2 | 5976.712461 | 0.000397614 | 53 |
| IGF1R | 2205.634 | 0.000438982 | 125 |
| SHH | 2489.204333 | 0.000433088 | 103 |
| TXN | 4781.745674 | 0.000430108 | 103 |
| MET | 3860.470936 | 0.000434216 | 109 |
| THY1 | 2383.869928 | 0.0004329 | 111 |
| KLF4 | 3433.88044 | 0.000424088 | 88 |
| NFKBIA | 3328.238164 | 0.000454752 | 174 |
| MAPK14 | 6019.020017 | 0.000455996 | 177 |
| CAT | 11323.96642 | 0.000458295 | 183 |

| | | | |
|---|---|---|---|
| PLCG1 | 3038.392761 | 0.000421763 | 94 |
| MDM2 | 2413.374066 | 0.00043802 | 116 |
| PDGFRB | 2962.560662 | 0.000433651 | 130 |
| HSP90B1 | 2000.20075 | 0.000430849 | 91 |
| FOS | 13218.43916 | 0.000471698 | 223 |
| CYCS | 12686.69332 | 0.000456204 | 183 |
| TERT | 2794.018925 | 0.000419463 | 82 |
| PRKDC | 5000.423991 | 0.000404858 | 58 |
| CALR | 3066.366218 | 0.000431406 | 98 |
| WT1 | 1993.314268 | 0.000407166 | 55 |
| MAPT | 4743.638569 | 0.000434783 | 100 |
| MEF2C | 1652.115991 | 0.000401929 | 57 |
| STAT5A | 2259.69822 | 0.000432152 | 136 |
| SMAD4 | 3393.807963 | 0.000438404 | 117 |
| RAC1 | 2099.996896 | 0.000426439 | 98 |
| STAT1 | 4359.905006 | 0.000448229 | 182 |
| BECN1 | 3846.756714 | 0.000440917 | 124 |
| PARP1 | 4688.261252 | 0.000427899 | 100 |
| RUNX2 | 1724.075355 | 0.000432152 | 107 |
| ITGB3 | 4593.418532 | 0.00043573 | 128 |
| ALOX5 | 1628.708134 | 0.000398883 | 57 |
| FOXO3 | 1736.805065 | 0.000440141 | 132 |
| JAK2 | 3012.559813 | 0.00044287 | 154 |
| SQSTM1 | 2494.92734 | 0.000434594 | 111 |
| PRKCD | 4237.042697 | 0.000432713 | 93 |
| YWHAZ | 3581.603344 | 0.000428816 | 91 |
| LYN | 1963.876253 | 0.000426985 | 107 |
| CDKN2A | 3606.080462 | 0.000448029 | 141 |
| SHC1 | 1626.759047 | 0.000429369 | 111 |
| ENO1 | 2547.812037 | 0.000408831 | 59 |
| PKM | 1650.943991 | 0.000412031 | 63 |
| FOXO1 | 1922.933105 | 0.00043802 | 121 |
| LDHA | 2345.87093 | 0.000411353 | 65 |
| SIRT3 | 2331.115661 | 0.000421585 | 74 |
| VTN | 2225.313341 | 0.000421408 | 91 |
| KNG1 | 6384.70374 | 0.000423549 | 106 |
| REN | 5200.363827 | 0.000438404 | 113 |
| ITGA5 | 2558.319682 | 0.000405186 | 76 |
| LRRK2 | 5673.343885 | 0.000436681 | 108 |
| AGT | 2730.689095 | 0.000430849 | 100 |
| CTGF | 2096.05779 | 0.00043535 | 125 |
| CYBB | 5492.315127 | 0.000441891 | 126 |
| EDN1 | 6221.053366 | 0.000450653 | 153 |
| NOS1 | 2509.696544 | 0.000422476 | 72 |

| | | | |
|---|---|---|---|
| GFAP | 4547.898592 | 0.000446229 | 110 |
| GSK3B | 3320.310766 | 0.000449843 | 139 |
| ENO2 | 1689.015572 | 0.000423191 | 68 |
| CALCA | 2769.90922 | 0.000410846 | 66 |
| BDNF | 8844.262538 | 0.000463822 | 183 |
| NGF | 7665.161579 | 0.000461042 | 172 |
| TAC1 | 7216.192919 | 0.000427716 | 100 |
| HSPG2 | 3796.309235 | 0.00039032 | 67 |
| TXNRD1 | 1704.399196 | 0.000397456 | 65 |
| MYH6 | 3569.57079 | 0.000408163 | 57 |
| GRIA1 | 4338.468093 | 0.000411353 | 67 |
| GRIN2B | 5253.971137 | 0.000423729 | 79 |
| TIMP1 | 2775.446059 | 0.000440141 | 137 |
| MMP2 | 4489.116716 | 0.000454752 | 176 |
| PPIF | 5123.237374 | 0.00040404 | 63 |
| CASP1 | 2682.690356 | 0.00043573 | 135 |
| UBA52 | 21298.44203 | 0.000436681 | 127 |
| COL1A1 | 3619.640886 | 0.000421941 | 101 |
| COL3A1 | 1922.916436 | 0.000403551 | 78 |
| PRKCA | 7838.506168 | 0.000442478 | 114 |
| CXCL8 | 8660.91541 | 0.000481928 | 270 |
| P4HB | 4784.864075 | 0.000420875 | 83 |
| THBS1 | 5014.432665 | 0.000433839 | 127 |
| VCAN | 1785.457703 | 0.000397298 | 59 |
| SERPINE1 | 3637.909248 | 0.000443656 | 148 |
| VWF | 7158.704428 | 0.000443066 | 156 |
| ITGA2B | 2264.764853 | 0.000410341 | 87 |
| PLG | 5084.760086 | 0.000439174 | 134 |
| F3 | 1838.489213 | 0.000414938 | 86 |
| IGF1 | 8891.622257 | 0.00047081 | 231 |
| LOX | 2258.267308 | 0.000434594 | 106 |
| SPARC | 2020.053606 | 0.000411523 | 72 |
| ENTPD1 | 4063.021868 | 0.000382995 | 62 |
| CRH | 1645.042403 | 0.00040032 | 60 |
| CCK | 2738.393125 | 0.000396668 | 60 |
| GRM5 | 3216.048852 | 0.000393236 | 55 |
| ADRB2 | 1787.619939 | 0.000412371 | 60 |
| TRPV1 | 7134.836535 | 0.000423191 | 75 |
| AIF1 | 1691.685406 | 0.000425713 | 91 |
| ARRB1 | 3100.533003 | 0.000422119 | 78 |
| IL4 | 3233.191109 | 0.000457666 | 198 |
| CXCL12 | 2659.009239 | 0.000448833 | 185 |
| S100B | 1650.104684 | 0.000412541 | 54 |
| HMGB1 | 2112.393424 | 0.000435161 | 111 |

| | | | |
|---|---|---|---|
| MYD88 | 2191.009277 | 0.000434028 | 139 |
| ITGB2 | 1638.822404 | 0.000414079 | 102 |
| RAB7A | 3033.182344 | 0.000407498 | 58 |
| TGFB1 | 5499.036955 | 0.000451467 | 165 |
| CTSD | 3319.500446 | 0.000422297 | 76 |
| MAP2K1 | 3002.358626 | 0.000440335 | 120 |
| SPP1 | 3061.103698 | 0.000441891 | 147 |
| DNM2 | 3378.016519 | 0.000404204 | 52 |
| BMP4 | 3161.46007 | 0.000424989 | 104 |
| HMOX1 | 6640.549658 | 0.00045106 | 147 |
| IFNG | 2498.608477 | 0.000453309 | 178 |
| SOD1 | 5565.115585 | 0.000438982 | 128 |
| EEF2 | 1962.598855 | 0.000406669 | 57 |
| HSPA5 | 8597.355019 | 0.000453104 | 146 |
| IL17A | 2033.570434 | 0.000442674 | 158 |
| GAD1 | 2338.427461 | 0.000401284 | 61 |
| GDNF | 2910.140722 | 0.000422297 | 71 |
| ITGAX | 2288.336814 | 0.000424989 | 137 |
| NOD2 | 3802.253319 | 0.000403063 | 84 |
| CASP8 | 5741.156096 | 0.000448833 | 164 |
| FLT1 | 1600.976095 | 0.000431406 | 98 |
| EPRS | 4298.900279 | 0.000413052 | 68 |
| LMNA | 2909.850692 | 0.000427533 | 79 |
| SOD2 | 3474.461048 | 0.00043122 | 111 |
| HSPD1 | 2218.702113 | 0.00042337 | 84 |
| PTGS1 | 2666.559804 | 0.000396825 | 53 |
| IL13 | 2042.910625 | 0.00042735 | 141 |
| HGF | 2952.976403 | 0.000444642 | 149 |
| VCP | 1931.026733 | 0.000410341 | 62 |
| CDH5 | 2331.766827 | 0.00042517 | 92 |
| IL2 | 3932.546071 | 0.000455581 | 182 |
| FAS | 2198.736989 | 0.000419111 | 101 |
| CD40 | 2140.783168 | 0.0004329 | 156 |
| CCNB1 | 2014.052326 | 0.000417362 | 85 |
| DNM1L | 2780.633928 | 0.00040016 | 65 |
| MAP2K4 | 1719.401212 | 0.000398406 | 52 |
| B2M | 2693.709529 | 0.000429 | 102 |
| SP1 | 1835.680155 | 0.000427168 | 92 |
| MAPK9 | 2365.618075 | 0.000421053 | 81 |
| IKBKG | 2215.955772 | 0.000421408 | 91 |
| XBP1 | 3651.276178 | 0.000418585 | 76 |
| ETS1 | 1732.381093 | 0.00041425 | 70 |
